# Epidemiological Study of Traumatic Brain and Spinal Injuries in a Pediatric Population: A One-Year Analysis of Prevalence, Causes and Trends

**DOI:** 10.7759/cureus.57224

**Published:** 2024-03-29

**Authors:** Svetoslav Todorov, Petar-Preslav Petrov, Zlatko Kirovakov, Plamen Penchev

**Affiliations:** 1 Neurological Surgery, University Multi-profile Hospital for Active Treatment (UMHAT) "Burgas", Burgas, BGR; 2 Anatomy, Histology and Embryology, Medical University of Plovdiv, Plovdiv, BGR; 3 Healthcare, University "Asen Zlatarov", Burgas, BGR; 4 Medicine, Medical University of Plovdiv, Plovdiv, BGR

**Keywords:** trauma, pediatric, epidemiology, spinal cord injury, traumatic brain injury

## Abstract

Background

Traumatic brain injury (TBI) and spinal cord injury (SCI) are leading causes of morbidity and mortality in pediatric patients. However, the epidemiology of pediatric brain and spine injuries in Bulgaria is poorly documented. This study aims to analyze and identify the prevalence, causes, and trends of traumatic brain and spinal cord injuries in pediatric patients during the period of 1st June 2022 to 30th June 2023.

Methods

A retrospective study was conducted on the medical records of patients under 18 years of age who visited the emergency department of University Multiprofile Hospital for Active Treatment (UMHAT) Burgas, Bulgaria between 1st June 2022 and 30th June 2023. The incidence and etiology were stratified by age, gender, and anamnesis. Data processing and analysis were performed with the statistical package IBM SPSS v. 26.0 (IBM Corp., Armonk, NY, USA), and graphical analysis with MS Office Excel 2016 (Microsoft, Redmond, WA, USA). Means ± standard deviation and 95% confidence interval were calculated. All p-values less than 0.05 were considered indicative of statistical significance.

Results

Data for patients aged <18 years, admitted to the emergency department (ED) of UMHAT Burgas, Bulgaria from 1st June 2022 to 30th June 2023 were analyzed (*n*=38504). Of these patients, 32% were children (*n*=13857). One hundred thirty-four (0.3%) of the pediatric patients were hospitalized in the neurosurgical ward, and 4653 (10.7%) were hospitalized in other wards. Of the analyzed patients, 89 are boys (66.4%), 45 are girls (33.6%) (male-female ratio 2:1) and the mean age of the patients with a head trauma was 8.07 years old. The average number of patients by diagnosis is 13.4±35.37. The largest percent are patients with brain concussion (85.07%, n=114), followed by contusion of the nerve roots in the lumbar region or late contusion wound of the head (with 2.99% each, n=4); hydrocephalus or skull fracture (with 2.24% each, n=3); contusion of the nerve roots in the thoracic region (1.49%, n=2); and fracture at Th9 vertebrae, fracture at C2 vertebrae, brain trauma or brain tumor (with 0.75% each, n=1). The average number of patients by anamnesis is 13.2±17.99. The largest percent are patients who fall from their own height (44.78%, n=60); followed by falls from height (20.90%, n=28); car accident (7.46%, n=10); injured by fight, fall from a bicycle or incident during a football game (with 5.97% each, n=8); fall from electric scooter (4.48%, n=6); hit in the closet (2.99%, n=4); and finally from bike accident or hit by a rock (with 0.75% each, n=1). From 134 hospitalized patients in neurosurgery, 114 (85.07%) did not require surgical treatment and were treated with conservative treatment and 20 (14.93%) were treated surgically.

Conclusion

In conclusion, this study highlights a significant burden of pediatric traumatic brain and spinal injuries in Bulgaria. The majority of cases were managed conservatively, emphasizing the need for preventive measures.

## Introduction

Traumatic brain injury (TBI) is the primary cause of death and disability in children. TBI results in a range of injuries to the scalp, skull, and brain that are comparable to those in adults but differ in pathophysiology and management due to age-related structural changes, injury mechanisms based on the physical abilities of the child, and the challenges in the neurological evaluation of pediatric populations. The scalp is highly vascularised and can cause lethal blood loss. Even a small loss of blood volume can lead to hemorrhagic shock in a newborn, infant, or toddler, which may occur without apparent external bleeding [1]. 

Spinal cord injuries (SCIs) in children present unique and complex challenges for clinicians, researchers, and families. Each year, a significant number of children sustain SCIs due to various causes, including trauma, congenital malformations, and acquired diseases [2,3]. These injuries have profound implications for a child's overall health, functional abilities, and quality of life. Therefore, understanding the complexity of SCI in children and developing effective interventions is of paramount importance [4-6].

Pediatric spinal cord injury has several significant distinguishing features that are related to the tissue maturation stage of the child’s spinal cord and the disparity in size between the child and an adult. There is a variability in the structural and morphologic features of the growing pediatric spinal cord, and the changes in it differ after injury as well [7]. Safe for teenagers (adolescents) to participate in it, because it is more pliable and elastic, leading to different types of injuries and the potential to heal differently, compared to adults. Likewise, when we are drafting treatment strategies for children, we take into account that growing body to strike the balance of the best outcomes for the relevant functions while possible, and with minimal complications.

The primary cause of death in children is intentional injuries. Compared to all other traumatic injuries, brain injuries result in death or disabilities the most frequently. Several research studies conducted by the U.S. Centers for Disease Control and Prevention provide a huge wealth of data on TBI in children. The number is around 475,000 U.S. children aged 0-14 who suffer from TBI annually. From these studies, the figure has shown that up to 90% of these impacts do not influence severe injuries and people can go home. Nevertheless, 37,000 cases need hospitalization, and 2,685 people pass away as a result of their injuries [2,4,7].

The incidence of pediatric SCI varies depending on the population studied and the definition of SCI used. According to the National Spinal Cord Injury Statistical Center, SCI in children is relatively rare, accounting for less than 4% of the overall annual incidence of SCI [8]. In the United States, the annual incidence of SCI is approximately 54 cases per million, with a higher incidence in males than in females [8]. The incidence of SCI in pediatric populations has changed significantly over time. In adolescents, the incidence of SCI decreased from 13 per million people to eight per million between 1997 and 2012.

With age, the SCI cases dramatically increase over a short period. Incidents within the age range of 17-23 compose more than 30% of all injuries, yet individuals age 16-30 comprise nearly 53% of all injuries [9]. However, it is essential to mention that children heal and recover faster than adults do after injury. At different ages, the levels of SCI vary with C2 lesions in preteen groups being the most common, followed by teen groups having mostly C4 lesions and adults’ cases mostly C4-5 lesions [9].

The purpose of the study is to conduct a detailed epidemiological analysis that will focus on the pediatric population’s traumatic brain injuries and spinal cord injuries. The study intends to find out the percentage, causation, and recent developments in these significant injuries through a thorough examination during the year. This study will seek to provide the community with knowledge on the incidences of traumatic brain and spinal injuries among children by collecting and interpreting relevant data. The target is therefore to find remedies that can protect people from injuries such as these.

## Materials and methods

We conducted a retrospective study of medical records of patients <18 years of age who attended the emergency department of University Multiprofile Hospital for Active Treatment (UMHAT) Burgas, Bulgaria in the period 1st June 2022 to 30th June 2023. The incidence and etiology were stratified by age, gender, and anamnesis.

From a public health perspective, we conducted this study in the city of Burgas, which is the fourth biggest city in Bulgaria. It is home to 202,766 residents. The mean age of the population in Burgas is 42.9 years and children under the age of 19 comprise 41,224 residents of the population (20.33%). Depending on their location, major trauma cases are transferred to a trauma center. UMHAT Burgas, where the study was conducted, is one of the trauma centers in Burgas.

Data processing and analysis were performed with the statistical package IBM SPSS v. 26.0 (IBM Corp., Armonk, NY, USA) and graphical analysis with MS Office Excel 2016 (Microsoft, Redmond, WA, USA). Means ± standard deviation and 95% confidence interval were calculated. All p-values less than 0.05 were considered indicative of statistical significance.

## Results

**Figure 1:**
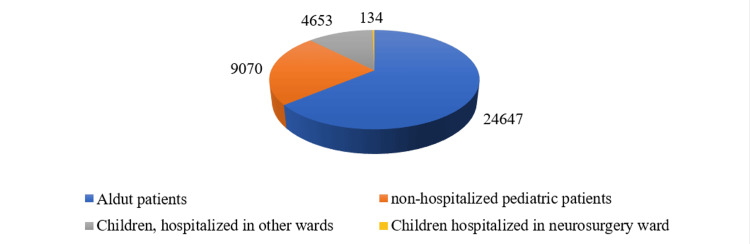
Distribution by children and adults. Тhe data has been represented as N values. All p-values less than 0.05 were considered indicative of statistical significance

**Figure 2:**
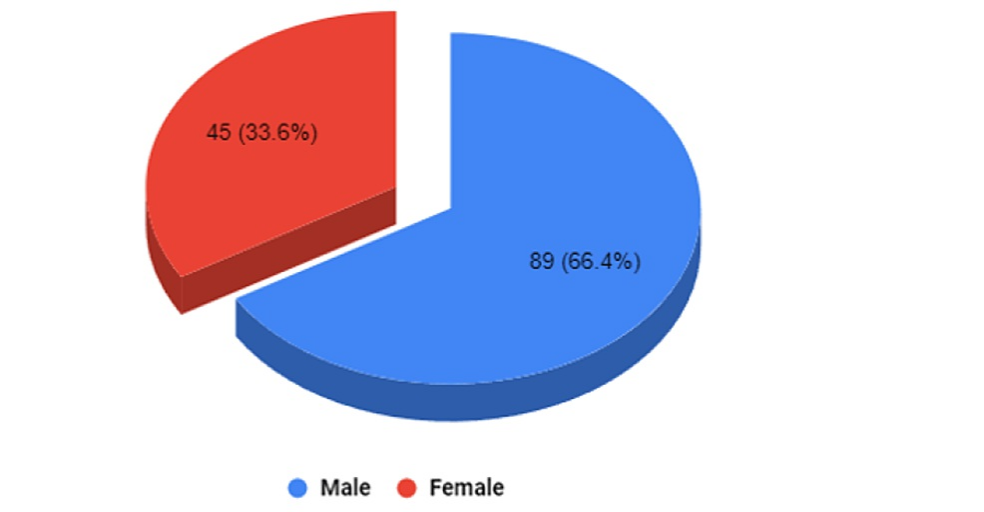
Distribution оf the patients hospitalized in the neurosurgery ward by gender. The data has been represented as N and % values. All p-values less than 0.05 were considered indicative of statistical significance

Data for patients aged <18 years, admitted to the emergency department (ED) of UMHAT Burgas, Bulgaria from 1st June 2022 and 30th June 2023 were analyzed (n=38,504). Of these patients, 32% were children (n=13,857). 0.3% of the pediatric patients (n=134) were hospitalized in the neurosurgical ward, and 10.7% (n=4653) were hospitalized in other wards (Figure 1, Table 1). Of the analyzed patients, 89 are boys (66.4%) and 45 are girls (33.6%) (male-female ratio 2:1) (Figure 2).

**Table 1:** Distribution of the admissions to the ED by children and adults

Total number of patients who attended the ED	38 504
From which children	13 857
Children, hospitalized in other wards	4 653
Children hospitalized in neurosurgery ward	134

**Figure 3:**
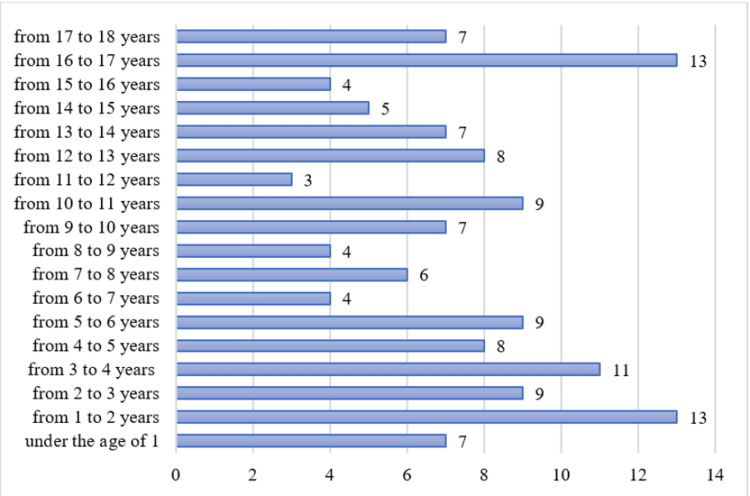
Distribution of the patients, hospitalized in the neurosurgery ward by age. The data has been represented as N values. All p-values less than 0.05 were considered indicative of statistical significance

The average number of patients hospitalized in the neurosurgery ward is 7.44±2.93. The distribution of the patients hospitalized in the neurosurgery ward by age is presented in Figure 3. The mean age of the patients who sustained a head trauma was 8.07 years old. The largest percent of patients is between one to two years and 16-17 years (with 9.7% each, n=13); after that patients between three to four years (8.21%, n=11); patients between two to three years, five to six years and 10-11 years (with 6.72% each, n=9); patients between four to five years and 12-13 years (with 5.97% each, n=8); patients under the age of one, and between nine to 10 years, 13-14 years and 17-18 years (with 5.22% each, n=7); patients between seven to eight years (4.48%, n=6); patients between 14-15 years (3.73%, n=5); and patients between six to seven years, eight to nine years and 15-16 years (with 2.99% each, n=4).

**Figure 4:**
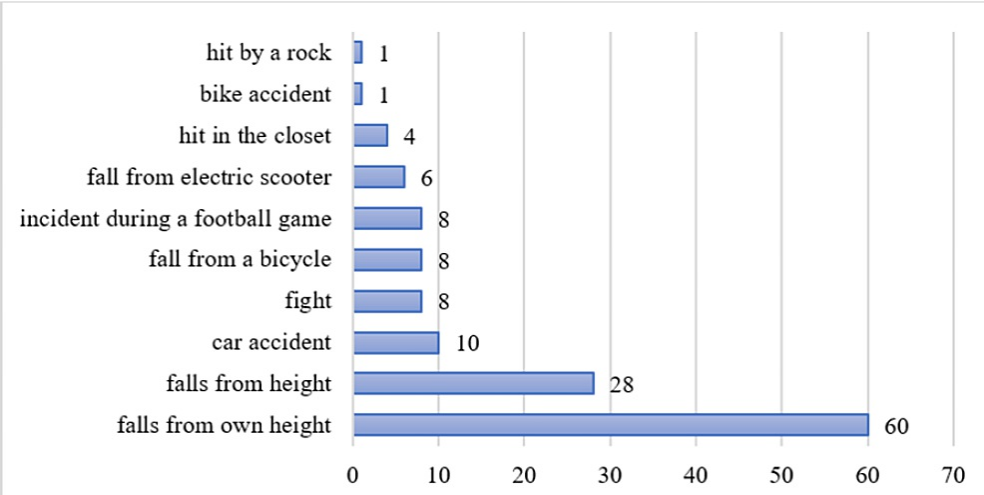
Distribution of the patients by anamnesis The data has been represented as N values. All p-values less than 0.05 were considered indicative of statistical significance

The average number of patients by anamnesis is 13.2±17.99. The distribution of the patients by anamnesis is presented in Figure 4. The largest percent are patients who fall from their own height (44.78%, n=60); followed by falls from height (20.90%, n=28); car accident (7.46%, n=10); injured by fight, fall from a bicycle or incident during a football game (with 5.97% each, n=8); fall from electric scooter (4.48%, n=6); hit in the closet (2.99%, n=4); and finally from bike accident or hit by a rock (with 0.75% each, n=1).

**Figure 5:**
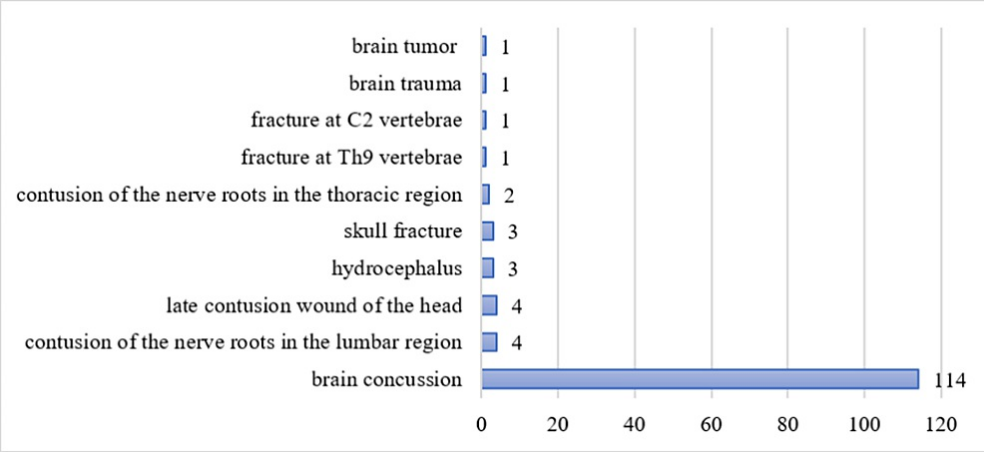
Distribution of the patients by diagnosis The data has been represented as N values. All p-values less than 0.05 were considered indicative of statistical significance

The average number of patients by diagnosis is 13.4±35.37. The distribution of the patients by anamnesis is presented in Figure 5. The largest percent are patients with brain concussion (85.07%, n=114); followed by contusion of the nerve roots in the lumbar region or late contusion wound of the head (with 2.99% each, n=4); hydrocephalus or skull fracture (with 2.24% each, n=3); contusion of the nerve roots in the thoracic region (1.49%, n=2); and fracture at Th9 vertebrae, fracture at C2 vertebrae, brain trauma or brain tumor (with 0.75% each, n=1).

From 134 hospitalized patients in neurosurgery, 114 (85.07%) did not require surgical treatment and were treated with conservative treatment, and 20 patients (14.93%) were treated surgically. Most cases were managed conservatively, drawing the need for preventive measures such as promoting safe play environments and responsible behavior, such as the use of helmets and seatbelts.

## Discussion

The head is the most commonly affected area in pediatric trauma [10-12]. A recent study published by Osmond et al. reported that head injury is the most common injury (65%) among children with injury severity score (ISS) > 11 following trauma in a study from Canada [10]. It is estimated that the incidence of TBI in the US is about 73.5 per 100,000 people. However, this figure may vary depending on the study methodology and reporting area, with some studies indicating even as high as 300 per 100,000 [11]. Ivan et al. reported a head injury rate of 8.8% among pediatric admissions in a Canadian trauma center [13].

Rohana et al. reported in a hospital-based study in Malaysia, that head injuries accounted for 4.75% of all cases in the emergency department [14]. A recent study from Nigeria of road traffic incidents involving children by Adesunkamni et al. found that head injury was the most common mode of injury [15]. In our study, head trauma was also the most common diagnosis, accounting for 114 (85.07%) of the cases, so we correlate with the findings of the studies above.

In our findings, the predictors associated with sustaining a head injury were male gender (n=89, 66.4%), age under 12, with the age one to two and 16-17 accounting for most of the cases (n=13, 9.07%). The mean age of the patients who sustained a head trauma was 8.07 years old. Our results correlate with other observational studies by Crankson et al. and Kapp et al. [16,17]. The male dominance (male-female ratio 2:1) was also reported in another study by Rickels et al. [18]. A study by Alhabdan et al. discovered that falls tend to dominate in younger age groups, and vehicle crashes tend to affect mainly older children and teenagers [19]. This could be associated with a tendency for high speed and reckless driving, as well as underage driving and inappropriate driver licensing, within this age group [19].

In our study, falls from own height accounted for most of the cases (n=60, 44.78%), and fall from height accounted for 28 cases (20.90%). Such findings can be found also in a study by Mazurek [20]. Tabish et al. report that such results can be explained by the fact that the cognitive, perceptual, and judgemental abilities of younger individuals are not fully developed [21]. This is also an indication of the importance of having safe outdoor play environments. Municipalities should prioritize the provision of safe parks for children to play in, which are more common in developed countries.

Approximately two-thirds of pediatric trauma admissions to trauma centers involve head injuries. Male gender, age under 12 years, and motor vehicle crashes are factors that correlate with head injury in children [22,23]. Nationwide interventions are needed to prevent injuries by promoting seat belt and helmet use and by educating against dangerous driving practices [24]. There is a need for more population-based data in our country and other emerging economies to inform policymakers and to be a driver for change.

Our study is limited in several ways. First, its retrospective nature makes it susceptible to multiple biases, such as information bias. Biases associated with retrospective analyses cannot be ignored, although the data collection, data cleaning, and quality control mechanisms of our trauma database are comparable to similar databases. Second, referral bias may be important because this is a hospital-based study. We need population-based data to effectively inform policy makers' decisions.

## Conclusions

In conclusion, this study highlights the significant burden of traumatic brain injury and spinal cord injury in the pediatric population in Bulgaria, especially in the catchment area of UMHAT Burgas. Various types of injuries, predominantly head trauma, with falls being the most common cause, occurred in the pediatric population with an average age of 8.07 years. The need for targeted preventive measures was highlighted by the male predominance and the correlation between age groups and the fact that most of the cases were managed by conservative treatment.

Our findings reinforce the importance of injury prevention strategies, particularly in promoting safe play environments and responsible behavior, such as the use of helmets and seatbelts. Although our study has limitations due to its retrospective design and hospital-based nature, it provides valuable insights into the epidemiology of childhood traumatic brain and spinal injuries in Bulgaria. 
